# Investigation of the Paediatric Tracheostomy Decannulation: Factors Affecting Outcome

**DOI:** 10.22038/ijorl.2019.37265.2217

**Published:** 2020-05

**Authors:** Neha Chauhan, Satyawati Mohindra, Sourabha K Patro, Preethy J Mathew, Joseph Mathew

**Affiliations:** 1 *Department of Otolaryngology and Head and Neck Surgery,* *Postgraduate Institute of Medical Education and Research, Chandigarh, India.*; 2 *Department of Anaesthesia, Postgraduate Institute of Medical Education and Research, Chandigarh, India.*; 3 *Department of Paediatrics, Postgraduate Institute of Medical Education and Research, Chandigarh, India.*

**Keywords:** Decannulation, Factors Affecting Decannulation, Paediatric Tracheostomy, Prolonged Mechanical Ventilation

## Abstract

**Introduction:**

Evidence for factors determining paediatric tracheostomy decannulation vary extensively; therefore, this prospective observational study aimed to investigate these factors.

**Materials and Methods:**

In total, 67 consecutive paediatric patients (<12 years old) who referred to the Department of Otolaryngology, (Postgraduate Institute Medical Education and Research),(Chandigarh), India, for decannulation were included and evaluated for contributing factors in this study. Parental counselling was performed, and informed consents were obtained from them. The patients underwent detailed work up including X-rays of airway/soft tissue neck (STN) and endoscopic assessment under anaesthesia for evaluating airway patency. Decannulations were attempted post assessment and followed up one month to classify decannulation as success or failure regarding the removal of the tracheostomy tube.

**Results:**

Totally, 61 patients out of 67 cases were successfully decannulated, whereas six children failed the decannulation. Moreover, the duration of tracheostomy (Pearson’s Chi-square 35.330, P=0.013), indication of tracheostomy (Pearson’s Chi-square 21.211, P=0.000), STN X-Ray (Chi-square 43.249, P=0.000), and bronchoscopic findings (Chi-square 67.000, P=0.000) were significantly associated with the outcome of decannulation. However, decannulation outcome had no significant correlation with various factors, such as the duration of intubation preceding tracheostomy, duration of ventilation, tracheal swabs, and antibiotic therapy.

**Conclusion:**

The STN X-ray is an independent predictor, and it is recommended for paediatric tracheostomy decannulation. Moreover, bronchoscopic assessment should be performed in children having doubtful infra-stomal airway. Duration of tracheostomy significantly affects decannulation outcome. However, intubation duration preceding tracheostomy and duration of assistive ventilation have no direct effects on the outcome of decannulation. In children, gradual decannulation should be preferred and one month follow up is adequate for deciding decannulation outcome.

## Introduction

Tracheostomy (an artificial airway) involves surgical creation of a stoma in skin that leads into the trachea. Initial descriptions of tracheostomy dates back to 100 B.C by Asclepiades with first documented successful paediatric tracheostomy in the early part of the 17^th^ century (1,2). Tracheostomy is the most common surgical procedure performed on critically-ill patients for prolonged airway and ventilatory support (3). Children compared to adults have increased technical difficulties during performing tracheostomy and post tracheostomy care with higher morbidity and mortality after discharge (3,4). Indications in children include prolonged mechanical ventilation, impaired neurological status, inability to cough excessive secretions, and acute/chronic upper-airway obstruction (5).

Decannulation involves the removal of the tracheostomy tube once the patient can breathe and protect his/her airway naturally. Moreover, it improves voice and swallowing function, quality of life parameters, comfort, and perceived physical appearance (5); additionally, it makes discharge to home or another care facility easier.

There is a dearth of research regarding the pediatric tracheostomy decannulation outcome, and according to the literature of the last two decades (PubMed), the overall successful decannulation rates varied from 35% to 75% (6-13).Variations in population and practices make the interpretation of the rates and factors difficult for pediatric tracheostomy decannulation (11). Therefore, a prospective observational study was planned to decipher various factors associated with paediatric tracheostomy decannulation outcomes at Postgraduate Institute of Medical Education and Research, Chandigarh which caters to six states of the northern India.

## Materials and Methods

This prospective observational study was conducted at the Department of Otolaryngology-Head and Neck Surgery in association with departments of Anaesthesia and Paediatrics, PGIMER, Chandigarh, India. This study included 67 consecutive paediatric patients (<12 years of age) referred for tracheostomy decannulation under the senior author supervision during January 2014 to April 2015. It should be noted that parental counselling was performed, and informed consents were obtained from them.The patients with congenital syndromes were excluded since syndromic pre-existing anatomical and physiological factors might affect/prevent decannulation. Furthermore, they were assessed regarding tracheostomy indication, history and duration of prior intubation, duration of active ventilation, type of tube during intubation, cuff pressure, tracheostomy tube type (cuffed/uncuffed), tracheostomy tube size, time since tracheostomy, time taken for decannulation, and swallowing function for solids and liquids. It should be noted that demographic characteristics of the patients was taken along with their routine Head and Neck, Ear, Nose and Throat history, examination, and profile.

Phonation was assessed in cooperative patients using stroboscopic evaluation for vocal cord and fold mobility/status. Cord mobility in younger children was assessed during general anaesthesia for endoscopic assessment with spontaneous mode of ventilation. This procedure involved the following steps in a sequential manner as sedation by intravenous/inhalational agent, evaluation of the vocal cords and larynx by fiber optic examination and laryngoscopy, administration of short acting muscle relaxants, rigid bronchoscopy for the evaluation of sub-glottis and suprastomal trachea, adequate ventilation with 100% oxygen and removal of tracheostomy tube, examination of stomal and infra stomal trachea up to carina and primary bronchus, reinsertion of the tracheostomy tube, wait for spontaneous ventilation to return, re-evaluation and confirmation of vocal cord mobility using laryngoscopy and fiber optic evaluation for ruling out any dynamic obstruction after weaning off from the effects of muscle relaxant and return of spontaneous ventilation in a sedated state, and awaking from sedation by withdrawal of the sedating agent after completion of the procedure. All patients were subjected to chest and soft tissue neck (STN) anteroposterior and lateral view X-rays. Moreover, they underwent bronchoscopic assessment under general anaesthesia to look for the patency of the airway/subglottic stenosis and presence of granulations or suprastomal ledge. Bronchoscopic assessments were followed with decannulation trial involving gradual downsizing of the tracheostomy tube, strapping over the tube, and removal of the tracheostomy tube. Each step was taken 48 h before proceeding to the next. Office based follow-up evaluation was conducted after one month of decannulation trial. Outcomes of decannulation, condition of stoma, as well as the status of swallowing, breathing, phonation, and cough reflexes were noted in this study. Patients with granulations or suprastomal ledges were managed with short course of systemic steroids and antibiotics followed by a repeat bronchoscopic assessment after 6 weeks. It is worth mentioning that all patients were followed up until the end of the study period. The obtained data were analyzed in SPSS software (version 20.0)(14).

## Results

In total, 67 patients including 22 and 45 females and males with the mean ages of 3.36±3.05 and 5.61±3.83 years, respectively, participated in this study from January 2014 to April 2015. Out of these cases, three males and three females failed decannulation. Moreover, age had no significant effect on decannulation of tracheostomy tubes. Due to various pre-existing pathologies, 60 patients required tracheostomy for prolonged mechanical ventilation, five patients required tracheostomy for acute upper airway obstruction due to acquired causes and two patients ended up with tracheostomy due to congenital airway narrowing. Out of the five patients who required tracheostomy due to acquired causes, two cases had retropharyngeal abscess, and one each had faucial diphtheria, Juvenile onset respiratory papillomatosis, and Ludwig’s angina. Of the two patients, who underwent tracheostomy for congenital narrowing of airway, one had congenital subglottic stenosis and one child had suspected congenital subglottic stenosis with laryngomalacia.

The indications for tracheostomy had significant effects on the outcome of decannulation (Pearson’s Chi-square 21.211, df2, P=0.000) with higher proportional chance of a failed decannulation in cases of airway narrowing/stenosis and gradually reduced chances of a failed decannulation for those tracheostomised for prolonged mechanical ventilation and non intubatable acute airway obstruction in a decreasing order. A total number of four out of 60 patients, who were tracheostomised for prolonged mechanical ventilation, and both the patients tracheostomised for narrowing of the airway failed decannulation. In the same line, 49 out of 67 patients had a duration of tracheostomy as less than 6 months (2/49 failed decannulation), and no patients had tracheostomy for less than 4 weeks. Similarly, 10 patients were tracheostomised for >6 months but <12 months (2 out of 10 cases failed decannulation), and 8 cases were tracheostomised for at least a year before decannulation (2 out of 8 cases failed decannulation). Duration of prior tracheostomy was significantly associated with the outcome of decannulation. There were increased chances of failure of decannulation with prolonged duration of tracheostomy (Pearson’s Chi-square 35.330, df 19, P=0.013).Out of the patients, six cases were never intubated before undergoing tracheostomy, and of these, four ones had acute respiratory obstruction and two cases had airway stenosis as the indication of tracheostomy. Regarding the intubation duration, 26 patients had prior intubation duration for ≤7 days, whereas others were intubated for >7 days. According to the results, the outcome of trial of decannulation was not found to be significantly associated with duration of prior intubation (Chi-square 14.461, df 11, P=0.209). Similarly, duration of ventilation had no significant association with outcome of decannulation (Chi-square 15.731, df 22, P=0.829).

Totally, four patients had narrowing of the airway and 60 cases had normal subglottic airway in STN X-ray. It should be noted that all these patients failed decannulation. On bronchoscopic evaluation, 61 subjects had normal airway, one patient had collapsible airway (Laryngo-tracheomalacia) with normal X-ray findings, and five cases had stenosis in the airway with subglottic involvement in four patients (Grade III stenosis in 3 patients and Grade IV stenosis in 1 case) followed by supra glottic involvement at the level of the epiglottis and Aryepiglottic fold in one patient (with normal sub glottic airway in X ray). The patient with supra glottic stenosis was later planned for LASER release of stenosis. Additionally, both X ray findings (Chi-square 43.249, df 1, P=0.000) and bronchoscopic assessment (Chi-square 67.000, df 6, P=0.000) findings were significantly correlated with the outcome of the decannulation process.

In bronchoscopic examinations, out of 61 patients with no fixed stenosis, 24 cases had granulations in the supra stomal airway, and others had normal subglottic airway (Fig’s 1,2). 

**Fig1:**
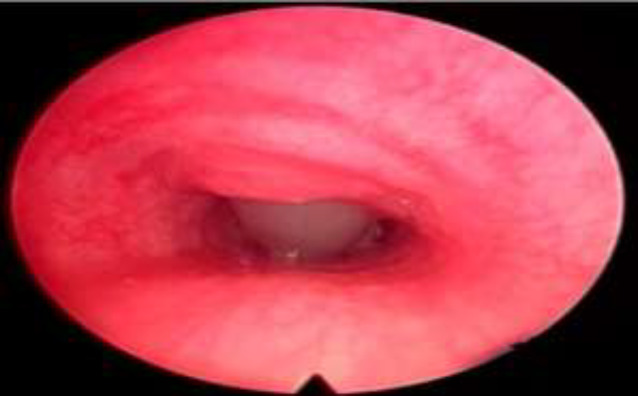
Endoscopic view of trachea with tracheostomy tube in situ

**Fig 2:**
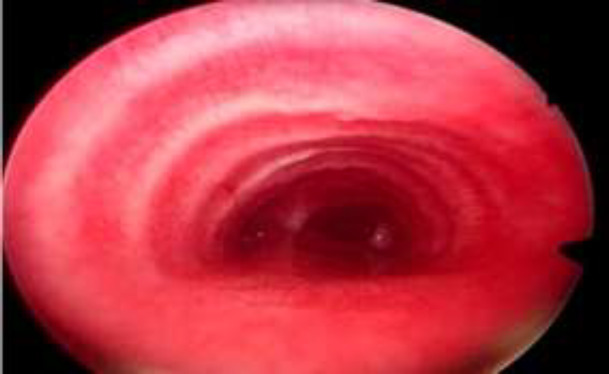
Endoscopic view of trachea till carina

Moreover, out of these 24 patients, granulations were minimal (<50% of the lumen) in 21 cases and occupied >50% of the lumen in three subjects (Fig’s 3,4). However, interestingly, all these 24 patients were successfully decannulated. 

**Fig 3:**
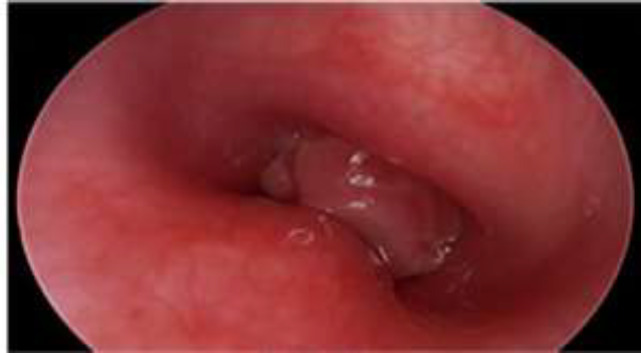
Suprastomal granulations occupying >50% of tracheal lumen

With respect to tracheal cultures, 25 patients had positive tracheal cultures, of which only one case failed decannulation, and five patients 

**Fig 4:**
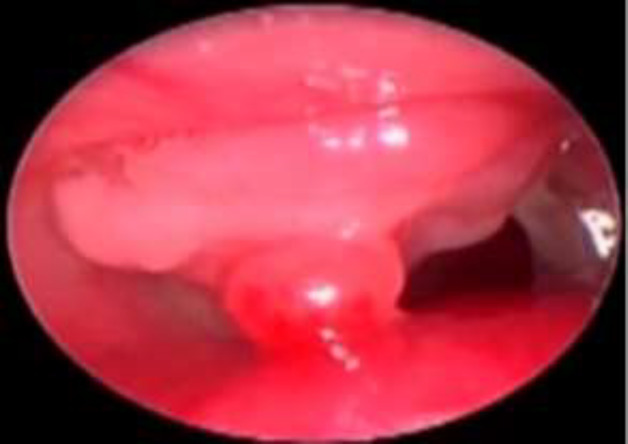
Suprastomal granulations < 50% of lumen

**Fig 5:**
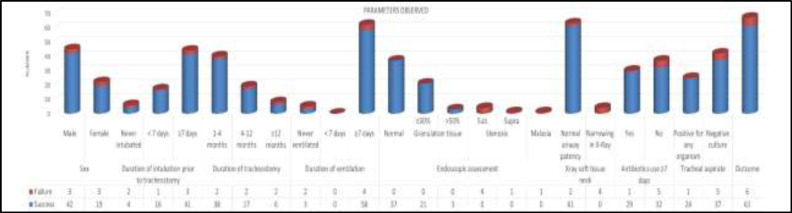
Graph showing various observed factors

Who failed decannulation had negative tracheal cultures. Various isolated organisms were Acinetobacter sp., Methicillin-Resistant Staphylo- coccus aureus, and Pseudomonas aeruginosa. Outcome of tracheal swab cultures had no significant effect on the outcome of the decannulation (Chi-square 2.302, df 4, P=0.680). Similarly, antibiotic therapy had no significant statistical correlation with the outcome of decannulation (Chi-square 13.962, df 9, P=0.124). Figure 5 depicts various factors observed in this study.

## Discussion

In the present study, tracheostomy duration, type/size of endotracheal tube, and duration of ventilation had no significant association with the outcome of decannulation. This led to reassess the variables influencing paediatric tracheostomy decannulations.

Chest X-ray (CXR) was performed for all patients before referral which highlighted the CXR to be the preferred basic investigation by most physicians. Moreover, STN X-ray, endoscopic assessment findings, duration of tracheostomy, and indication of tracheostomy were associated significantly with the decision for decannulation. Therefore, these techniques should be considered for making deciding on decannulation in future. Furthermore, neurological status and ability for airway protection should be considered before making decision on decannulation. Endotracheal/tracheostomy tube type, tracheal swab cultures, prolonged antibiotic usage, and duration of ventilation should also be considered. However, no significant association was noted between the utilization of these techniques and the decision for decannulation in the present study among the Indian population. This result is inconsistent with the earlier evidence in literature which suggested the association between these factors and subglottic narrowing affecting the outcome of decannulation. Tracheostomy decannulation is associated with a great risk because for cases who cannot be ventilated with bag and mask or endotracheal tube, there exists risks of losing the airway in emergent situations. 

Currently, decannulation failure has no accepted definition (15). In the present study, any patient requiring reinsertion of artificial airway within a period of 1 month of decannulation was considered as decannulation failure. Total observed failure rate was determined at 8.95% in this study. In contrasts to the results in this study, retrospective analysis of 119 paediatric tracheostomised patients treated over 30 years by de Trey L et al. demonstrated that airway obstruction was the most common indication for tracheotomy 70%) followed by prolonged mechanical ventilation (30%) (11). 

They showed serious complications in 25 patients (23%), tracheostomy related death in one patient, and successful decannulation in 60% of the cases(11). Moreover, Carron JD et al. found 3.2+/-0.6 years as the mean age of tracheotomy in their retrospective chart review (12), and they classified indications for tracheostomy into six groups, namely neurological impairment (27%), prolonged intubation (26%), obstruction of upper airway (19%), craniofacial abnormalities (13%), paralysis of vocal fold (7%), and trauma (7%). 

In the aforementioned study, the rate of successful decannulation was 41%, and time to decannulation was shorter in the craniofacial group than in those with neurological impairment and prolonged intubation groups. In addition, complication, tracheotomy-related death and overall mortality rates were 44 %, 3.6%, and 19%, respectively. Leung R et al. in their retrospective chart review identified patient diagnosis and tracheostomy indication as significant predictors for the duration of cannulation (16). Significantly shorter durations of cannulation were observed in patients who underwent tracheostomy for tracheobronchial toilet than those with neurological and traumatic indications. In the same vein, Simma B et al.(17) performed a review of the records of 108 patients for a period of 10 years and reported the indications for tracheostomy as acquired tumors (11.1%), paralysis of bilateral vocal cord (22.2%), congenital airway malformations (22.2%), and subglottic stenosis (31.4%). Their study documented a successful decannulation in 85 out of 108 patients (78.7%) and median period of tracheostomy of 486 days (8 days, 6.6 years). In the present study, prolonged mechanical ventilation and pulmonary toileting were the most common indications for paediatric tracheostomy comprising of 89.56% of the patients followed by acute airway obstruction in 7.46% and narrowing of the airway in 2.98% of them. Successful decannulation could be achieved in 91.04% of the children in our series in contrast to other studies (11,12,17). The mean age at tracheostomy in the present study was 4.88±3.70 years which was higher than that reported by Carron et al.(12). 

There was no tracheostomy related deaths in the present study, and decannulation rate was higher in prolonged intubation group (93.33%). Moreover, the longitudinal analysis showed an increase in the number of tracheostomies performed for prolonged intubation/ventilation and a decreasing trend in tracheotomy related complications(11).

Perin et al. evaluated the parameters affecting decannulation in head injury patients(18). They found higher rates of successful decannulation in patients of head trauma and spontaneous cough than those who had anoxic brain damage and presence of secretions. The association between the type of tracheostomy tube and outcome of decannulation in their study was similar to that in the present study; however, it is in contrast to the findings in other studies in literature.

A valid and unprovoked cough was identified as a useful parameter for successful decannulation. Such kind of evaluation of cough was not planned in our study since all patients were neurologically normal and able to protect their airway; however, in patients with neurological damage, the evaluation of cough, vocal cord mobility, and ability to protect airway play a significant role in making decision on decannulation from tracheostomy.

This dictum can be evidenced by the retrospective chart review conducted by Takahashi et al. on 42 patients of paediatric tracheostomy evaluating the success rate of decannulation in presence or absence of underlying diseases (19), indications for tracheostomy, degree of motor development (capable of walking unassisted), and ability to orally ingest food.

Out of 42 patients, only 11 cases were successfully decannulated in the mentioned study.

Singh et al. carried out a systematic review of tracheostomised patients aged 18 years and above and assessed the correlation of decannulation with swallowing and coughing (20). They designed a protocol of gradual downsizing and blocking in patients with long duration of mechanical ventilation while corking directly in patients with short duration of ventilation. 

In a study conducted by Sachdeva et al.(21), pre decannulation flexible fibre optic bronchoscopy was investigated in children with tracheostomy to identify possible causes of decannulation failure and intervention decision in 49 patients with mean duration of tracheostomy of 8 months. They found abnormal findings in 36/49 patients, the most common of which was airway granulation (51%). In total, 23 (46.9%) patients were successfully decannulated without intervention, whereas 15 (30.6%) cases needed interventions before attempting decannulation. In contrast, the present study reported granulations in 24/67 patients, and among these, 3 cases had granulations occupying more than 50% of the lumen. However, none of our patients required any further intervention, and all these patients were successfully decannulated.

In the same vein, Maslan et al. conducted a retrospective review on 188 patients aged up to 18 years and showed extremely low failure rate in only one (2.2%) patient out of 46 cases(22), who had institution based decannulation among 188 studied subjects. The patients with failures required reinsertion after 5 days of decannulation due to the intolerance of secretions. They used uncapped sleep study, direct laryngoscopy, rigid bronchoscopy, and sleep endoscopy (when indicated) to guide decannulation. 

The results of a retrospective chart review carried out by Chen C H et al. on 46 neonates revealed congenital or acquired airway obstruction as the most common indication with subglottic stenosis being the most common (23). The median age of the newborns was 104.5 days, and they reported no difference between term and pre term infants in terms of indications or decannulation outcomes. The findings of the present study showed acquired airway obstruction as the most common cause of failure of decannulation in two patients with congenital and acquired (post intubation) subglottic stenosis and one case with supra-glottic stenosis (following the ingestion of corrosive) and malacia. However, the most common cause in the present study was prolonged mechanical ventilation. In this study, gradual decannulation was practiced in all paediatric patients which was an agreed technique by other studies (24,25).

## Conclusion

Clinical assessment by the treating clinicians bears paramount importance for deciding decannulation. Indication and duration of tracheostomy must be considered for predicting the outcomes. Moreover, bronchoscopic assessment and STN X-ray prior to decannulation correlated significantly with the outcome of decannulation as independent predictors; therefore, they should be regarded as a necessary part of the evaluation. In addition, gradual and staged decannulation is safe for paediatric patients. One month is sufficient for deciding the outcome of primary attempt of decannulation since none out of those decannulated required re-cannulation after one month. Variables, such as duration of prior intubation, duration of ventilation, tracheal swab results, and antibiotic therapy must be documented. However, predictive efficacies of these variables are doubtful.

## Ethical Considerations

This study involved humans and was performed as a part of the thesis of the first author under the mentorship of Dr. Satyawati Mohindra (Senior Author). Therefore, ethical clearance and approval was required and obtained. The approval letter is added as supplementary material with letter number 9431/PG-2Trg/2013/3473 dated 04^th^ March 2014.
